# Gene therapy of rare diseases as a milestone in medicine - overview of the field and report on initial experiences in Slovenia

**DOI:** 10.1186/s13023-025-03828-8

**Published:** 2025-06-05

**Authors:** Urh Grošelj, Marko Kavčič, Ana Drole Torkar, Jan Kafol, Duško Lainšček, Roman Jerala, Matjaž Sever, Samo Zver, Gregor Serša, Maja Čemažar, Primož Strojan, Aleš Grošelj, Mojca Žerjav Tanšek, Špela Miroševič, Simona Ivančan, Tomaž Prelog, David Gosar, Jasna Oražem Mrak, Matej Mlinarič, Sara Bertok, Jernej Kovač, Jana Kodrič, Saba Battelino, Marko Pokorn, Alojz Ihan, Janez Jazbec, Tadej Battelino, Damjan Osredkar

**Affiliations:** 1 Department of Endocrinology, Diabetes, and Metabolic Diseases, University Children’s Hospital, University Medical Centre Ljubljana, Bohoriceva 20, Ljubljana, 1000 Slovenia; 2 Department of Paediatrics, Faculty of Medicine, University of Ljubljana, Ljubljana, Slovenia; 3 Department of Medical Ethics, Faculty of Medicine, University of Ljubljana, Ljubljana, Slovenia; 4 Department of Paediatric Haematology and Oncology, University Children’s Hospital, University Medical Centre Ljubljana, Ljubljana, Slovenia; 5 Faculty of Medicine, University of Ljubljana, Ljubljana, Slovenia; 6 Department of Synthetic Biology and Immunology, National Institute of Chemistry, Ljubljana, Slovenia; 7 Centre for the Technologies of Gene and Cell Therapy (CTGCT), National Institute of Chemistry, Ljubljana, Slovenia; 8 Department of Haematology, University Medical Centre Ljubljana, Ljubljana, Slovenia; 9 Institute of Oncology Ljubljana, Ljubljana, Slovenia; 10 Department of Otorhinolaryngology and Cervicofacial Surgery, University Medical Centre Ljubljana, Ljubljana, Slovenia; 11 Department of Family Medicine, Faculty of Medicine, University of Ljubljana, Ljubljana, Slovenia; 12 Department of Paediatric Neurology, University Children’s Hospital, University Medical Centre Ljubljana, Bohoriceva 20, Ljubljana, 1000 Slovenia; 13 Clinical Institute of Special Laboratory Diagnostics, University Children’s Hospital, University Medical Centre Ljubljana, Ljubljana, Slovenia; 14 Child Psychiatry Unit, University Children’s Hospital, University Medical Centre Ljubljana, Ljubljana, Slovenia; 15 University Children’s Hospital, University Medical Centre Ljubljana, Ljubljana, Slovenia; 16 Department of Infectious Diseases, University Medical Centre Ljubljana, Ljubljana, Slovenia; 17 Institute of Microbiology and Immunology, Faculty of Medicine, University of Ljubljana, Ljubljana, Slovenia

**Keywords:** Gene therapy, Rare genetic diseases, Slovenia, CRISPR/Cas9, CAR-T cells, Cancer, Immune gene therapy, CTNNB1 syndrome, Interleukin 12, CTGCT

## Abstract

Gene therapy has transitioned from a long-awaited promise to a clinical reality, offering transformative treatments for rare congenital diseases and certain cancers, which have a significant impact on patients’ lives. Current approaches focus on gene replacement therapy, either in vivo or ex vivo, mostly utilizing viral vectors to deliver therapeutic genes into target cells. However, refining these techniques is essential to overcome challenges and complications associated with gene therapy to ensure long-term safety and efficacy. Slovenia has witnessed significant advancements in this field since 2018, marked by successful gene therapy trials and treatments for various rare diseases. Significant strides have been made in the field of gene therapy in Slovenia, treating patients with spinal muscular atrophy and rare metabolic disorders, including the pioneering work on CTNNB1 syndrome. Additionally, immune gene therapy, exemplified by IL-12 adjuvant therapy for cancer, has been a focus of research in Slovenia. Through patient-centred initiatives and international collaborations, researchers in Slovenia are advancing preclinical research and clinical trials, paving the way for accessible gene therapies. Establishing clinical infrastructure and genomic diagnostics for rare diseases is crucial for gene therapy implementation. Efforts in this regard in Slovenia, including the establishment of a Centre for Rare Diseases, Centre for the Technologies of Gene and Cell Therapy, and rapid genomic diagnostics, demonstrate a commitment to comprehensive patient care. Despite the promises of gene therapy, challenges remain, including cost, distribution, efficacy, and long-term safety. Collaborative efforts are essential to address these challenges and ensure equitable access to innovative therapies for patients with rare diseases.

## Introduction

After several decades during which gene therapy was thought to be “just around the corner,” scientists have now succeeded in developing safe and clinically effective drugs for treating some rare congenital diseases and certain cancers. These advancements are making a significant difference in the lives of patients in Slovenia as well [1, 2].

Since 2018, when the first Slovenian child with a rare inborn metabolic disease was referred abroad for a gene therapy trial, several other children with various rare diseases have undergone genetic treatment [1, 2]. Slovenia’s approach to integrating gene therapy into clinical practice has some unique elements. The relatively small population and centralized healthcare system in Slovenia have facilitated a coordinated and rapid adoption of these therapies. For example, Slovenia’s first administration of CAR-T lymphocyte therapy in April 2020 marked a milestone achieved through collaboration between various national healthcare institutions, illustrating a streamlined and cohesive effort compared to the more fragmented healthcare systems in larger countries [3–5]. In November 2021, the first patient presenting basal cell carcinoma was enrolled in a phase I clinical study on immune gene therapy with interleukin 12 (IL-12) conducted jointly between the Institute of Oncology Ljubljana and the Department of Otorhinolaryngology and Cervicofacial Surgery of the University Medical Centre Ljubljana, showcasing the Slovenia’s proactive engagement in cutting-edge research. This is comparable to other countries’ efforts but is particularly notable given Slovenia’s smaller scale [5, 6]. Then, in December 2021, the first successful administration of gene therapy in Slovenia was conducted on a child with spinal muscular atrophy (SMA) at the University Children’s Hospital of the University Medical Centre Ljubljana (UCH), marking an important milestone for Slovenia, akin to larger nations but achieved with fewer resources [5, 7, 8]. Recently, there has been an intensive effort to develop this type of treatment within the Slovenia [1, 2, 6, 7, 9–13].

Simultaneously, there is extensive ongoing research in the field of gene therapy, with various treatments under development. As a result, we anticipate a significant expansion of these treatment options soon for numerous diseases that have previously lacked causal treatment. It is imperative that we, as a profession and as a society, are prepared for this and engage in public discussion, as new treatment modalities bring not only benefits but also numerous new challenges [14].

## Brief history of gene therapy

Gene therapy trials in humans began in the 1980s, but progress was hampered by adverse side effects and ethical concerns. In the gene therapy trials for X-linked severe combined immunodeficiency (SCID-X1), 5 of 20 patients treated with autologous bone marrow cells transduced with a retrovirus encoding the common γ-chain of cytokine receptors developed leukaemia, raising substantial concerns about the safety of gene therapy. This was later attributed to the insertion of the therapeutic gene near the *LMO2* oncogene, causing unchecked growth of transduced cells. In addition to tumorigenesis, another safety issue arose in a trial for ornithine transcarbamylase (OTC) deficiency, where a patient died from a severe immune reaction to a recombinant adenoviral vector delivering the *OTC* gene [15–17]. It wasn’t until the new millennium that research in this field began to flourish and show more promising results. Safety concerns regarding gene therapy, an essential aspect of any new treatment being developed, have been addressed over the last decade. This period has witnessed a growing number of gene therapies proven to be safe and effective, now available to patients [14, 18].

In Europe, in 2012, the first gene therapy for lipoprotein lipase deficiency (Glybera, uniQure) obtained market authorization (while already in 2004 it obtained orphan drug status), an exceedingly rare metabolic disorder leading to severely elevated triglyceride levels and heightened risk of acute and recurrent pancreatitis. However, this drug was withdrawn in 2017 due to insufficient market interest, partly attributed to its high price. Nonetheless, its introduction spurred the development of other gene therapies [19, 20].

Meanwhile, after decades of research, gene therapy has already been awarded a Nobel Prize; the 2020 Chemistry Prize went to French scientist Dr Emmanuelle Charpentier and American Dr Jennifer Doudna, who, with their research teams, developed the method of so-called “genetic scissors” (CRISPR/Cas9) just over a decade ago. It is a method of targeted excision and replacement of defective genes, which will further accelerate the otherwise lengthy path of developing gene therapies from the laboratory to the patient [21, 22]. Just a decade after the discovery of CRISPR/Cas9, we already have the first clinically approved drug for correcting mutations in beta-thalassaemia, with many other drugs currently in clinical trials [23].

Twelve different gene therapies are currently approved by the European Medicines Agency (EMA). These treatments address rare forms of blindness, leukaemia, spinal muscular atrophy, and various inborn immune deficiencies. While this number may seem small compared to the approximately 8,000 known rare diseases, most of which have a genetic basis, it is expected to increase rapidly in the coming years [14].

### Which diseases can be treated with gene therapy?

Various congenital genetic diseases, such as metabolic disorders, spinal muscular atrophy, cystic fibrosis, severe congenital dyslipidaemias, congenital cardiomyopathies, congenital blindness, and many others, are predominantly rare conditions (affecting fewer than one in 2,000 individuals). These diseases typically manifest in childhood, significantly diminishing the patient’s quality of life and shortening their lifespan. Moreover, they impose a substantial burden on patients, their families, and society at large. While many of these diseases currently lack effective symptomatic treatments, gene therapies have emerged as a promising approach for some conditions, offering the potential for successful causal treatment [24].

Gene therapy is typically aimed at treating diseases where the cause is a pathological change (mutation) in a single gene (out of over 20,000 genes comprising the human genome) [25]. Currently, gene therapy is not feasible for genetically inherited diseases that are polygenic or involve chromosomal abnormalities (changes in the number or organization of chromosomes) [26]. Additionally, the treatment with chimeric antigen receptor (CAR) T lymphocytes enables targeted therapy for certain types of cancer, irrespective of the combination of mutations that led to the development of the malignant condition [27].

### How does gene therapy work?

There are several approaches to gene therapy. Gene therapy aims to achieve permanent expression of the introduced gene (known as a transgene), which replaces the function of the defective gene causing the disease. This aims to alleviate or cure the disease while minimizing the occurrence of side effects in patients. However, it’s crucial to acknowledge that gene therapy has occasionally been associated with risks and potential side effects [14, 28, 29].

In *in *vivo gene therapy, a modified viral vector containing the appropriate genetic sequence is introduced into the body to replace the patient’s defective gene. These viral vectors are engineered to be incapable of replicating independently, but they can infect the patient’s cells and introduce the correct genetic sequence into cells of various tissues [30].

*Ex *vivo gene therapy involves taking the patient’s hematopoietic stem cells, maintaining them in culture in the laboratory, and then introducing the desired genetic sequence into them outside the body using a modified viral vector. The modified stem cells are then transferred back into the patient, where they distribute throughout the body, and the correct genetic sequence in these cells begins to replace the function of the defective gene in the patient’s cells. This approach to treatment has the potential to create a stable source of functional protein (e.g., enzyme) both in visceral organs (especially the liver) and in the central nervous system, depending on the disease being targeted [31].

The *ex *vivo approach necessitates complex manipulations to extract the patient’s cells, modify them, and then reintroduce them. In vivo targeting has garnered attention for its ability to circumvent the complexities associated with ex vivo manipulation, thereby reducing costs. However, significant advancements are required to enhance the efficiency of in vivo manipulation. Challenges such as the native and adaptive immune responses against delivery vectors, along with the consequences of off-target tissue editing, represent major hurdles that need to be addressed [32, 33]. The development of gene therapy, although based on a conceptually simple mechanism, entails complex and rigorous demands. The corrected gene must reach the appropriate tissues in the correct doses and at the right time (during the developmental period). Expression must remain stable over time and should not adversely affect the fundamental functioning of the tissue [30, 31, 34–36]. It is crucial to ensure the safety of gene therapy (following the paramount medical principle of “first do no harm” pioneered by Hippocrates). With some viral vector-based therapies, there may be transient adverse reactions associated with the introduction of the viral vector, but these can be well-managed and resolved over time [14, 37–39]. In principle, a single administration of gene therapy should be sufficient for a lifetime. However, definitive answers regarding long-term efficacy are still lacking, as these therapies have only been in use for a short period. It is possible that the effectiveness of some gene therapies may diminish over time [40, 41].

**Fig. 1:**
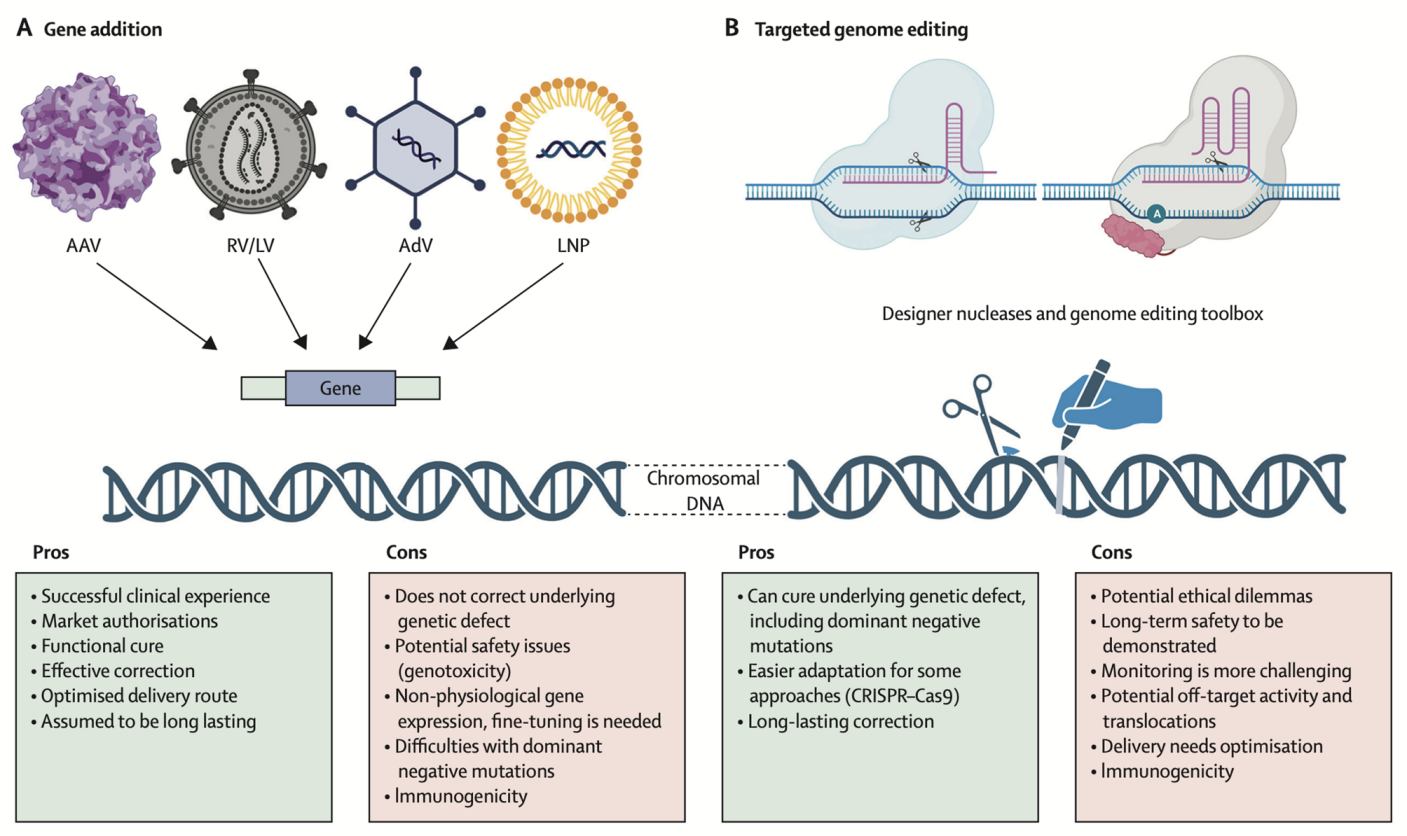
Gene addition approaches versus targeted genome editing approaches. (**A**) During gene addition, viral vectors, including AAV vectors, RV/LV vectors, AdV vectors, and non-viral vectors (e.g., LNPs), deliver a whole gene of interest with promoter or enhancer elements and polyadenylation signals. (**B**) Designer nucleases and the described genome editing toolbox (e.g., based on CRISPR–Cas9) lead to targeted gene editing with defined nucleotide changes in the genome. AAV = adeno-associated virus. AdV = adenoviral. Cas = CRISPR-associated protein. LNPs = lipid nanoparticles. RV/LV = lentiviral or gammaretroviral. Reprinted from The Lancet, Vol. 403, Axel Schambach, Christian J Buchholz, Raul Torres Ruiz, Klaus Cichutek, Michael Morgan, Ivana Trapani, Hildegard Büning, A new age of precision gene therapy, Pages 568–582, Copyright (2024), with permission from Elsevier

There are also several newer approaches to gene therapy that are not yet in clinical practice but hold promise for new treatments. One particularly promising approach is CRISPR-Cas9 gene editing (Fig. 1), which allows for precise correction of defects in a patient’s genetic material [14]. This approach holds promise for developing treatments for some widespread diseases such as cardiovascular disease by knocking out the *PCSK9* gene, a feat currently achieved with antibodies or RNA interference (RNAi) [42, 43]. In addition to gene replacement and correction, gene disruption is emerging as a powerful therapeutic strategy, particularly for gain-of-function disorders. A notable example is the successful in vivo CRISPR-Cas9–mediated disruption of the KLKB1 gene using lipid nanoparticle (LNP) delivery in patients with hereditary angioedema. This approach, employing NTLA-2002, achieved durable suppression of plasma kallikrein levels and significant reductions in disease attacks following a single intravenous dose, without severe adverse events [44].

LNPs have gained prominence as non-viral vectors for gene delivery due to several advantages over traditional viral vectors. They are non-immunogenic, reducing the risk of eliciting adverse immune responses, and are capable of encapsulating larger nucleic acid payloads, such as mRNA and plasmid DNA, which is particularly beneficial for delivering CRISPR components [45]. Furthermore, LNPs can be engineered for tissue-specific targeting, enhancing delivery efficiency and minimizing off-target effects [46]. Their modular design allows for rapid adaptation to different therapeutic needs, making them a versatile platform in the expanding field of gene therapy.

As a general rule, any gene therapy should be preferably administered before irreversible damage to the organism is caused by the disease, but this is often not possible in practice [36]. It is however becoming increasingly important to detect all diseases where such treatments are available as early as possible, ideally immediately after birth through universal newborn screening (in March 2024, Slovenia expanded newborn screening to include spinal muscular atrophy and primary immunodeficiencies, for which gene therapy is already or will soon be available) or through later screening tests in early childhood (this is how we detect familial hypercholesterolaemia in Slovenia, for which we also expect gene therapy to be available shortly, in the first phase especially for patients with the more severe, homozygous form of familial hypercholesterolaemia) [7, 47, 48].

The route of administration is a contributing factor in determining the effectiveness of gene therapy. Challenges have arisen following systemic delivery, including the limited ability of these therapies to penetrate the blood-brain barrier, particle degradation during transportation, heightened reactivity of the immune system, and toxicity in peripheral organs [49, 50]. To overcome these hurdles, both preclinical and clinical studies have proposed alternative routes of administration for gene therapies, with a focus on direct delivery into the cerebrospinal fluid (CSF). While certain CSF delivery methods may require invasive procedures, they have demonstrated significant clinical benefits by markedly enhancing the targeting of central nervous system cells compared to systemic intravenous delivery [51–53].

In the treatment of certain forms of cancer, the past decade has witnessed the rise of cellular immunotherapy. This approach goes beyond replacing a single defective gene; it involves genetically reprogramming the patient’s immune cells [54]. A subset of these cells, called T lymphocytes, are extracted from the patient’s blood using a process called apheresis. In the next step, they are genetically modified ex vivo in a specialized laboratory by introducing the genetic sequence for a CAR. These modified CAR-T lymphocytes are then transfused back into the patient, where they bind to a specific target molecule on the surface of cancer cells, proliferate, and trigger immune-mediated cell death of the cancer cells [27]. CAR-T cell therapy was initially approved for the treatment of children and young adults with acute lymphoblastic leukaemia and the treatment of adults with diffuse large B-cell lymphoma (DLBCL) in relapsed refractory setting after two previous lines of therapy. Currently, in the adult population, there are three anti-CD19 CAR-T cell products available for the treatment of various non-Hodgkin’s lymphomas, one product for acute lymphoblastic leukaemia and two anti-B-cell maturation antigen (BCMA) CAR-T cell products for treatment of multiple myeloma. We have seen the initial indications in advanced stages of disease being moved to a second line as is the case with axi-cell and liso-cell for DLBCL and cilta-cel for multiple myeloma. Several encouraging studies show that some groups of patients will benefit from CAR-T cell therapies in the first line setting in the near future [55–59]. On the other hand, clinical trials show that this approach could also be used to treat autoimmune diseases such as lupus and multiple sclerosis [60, 61]. Slovenia is making significant efforts to establish in-house CAR-T cell manufacturing and treatment capabilities through a collaboration between the Department of Haematology at the University Medical Centre Ljubljana and the Institute of Microbiology and Immunology at the Faculty of Medicine, University of Ljubljana. The collaboration led to the construction of cleanroom facilities, acquisition of CliniMACS Prodigy^®^ (Miltenyi Biotec, United States of America) machines for CAR-T cell production, navigation through the regulatory process, and is currently in the stage of submitting a clinical protocol for the treatment of acute lymphoblastic leukaemia in adults. Further indications for CAR-T cell therapy, which are not currently available in Slovenia, are planned to be addressed, and clinical protocols submitted. Collaboration is also being established with the National Institute of Chemistry (NIC) and the Centre for the Technologies of Gene and Cell Therapy (CTGCT) to develop their vectors for additional indications and safer usage [62, 63].

### Previous experience in Slovenian patients

In the field of paediatric neurology, SMA was the first disease to be treated with gene therapy using the drug onasemnogene abeparvovec. The first Slovenian child received this treatment abroad in 2019, and since December 2021, four more children have been treated at the UCH [7]. This treatment uses a modified viral vector (adenovirus-associated viral vector 9 [AAV9]), which infects cells in the human body and delivers a transgene that replaces the action of the defective gene responsible for the disease. It is crucial for the virus to reach the central nervous system, where the presence of the transgene is essential to halt disease progression [64]. All Slovenian children tolerated the treatment well, except for one who developed thrombotic microangiopathy during the treatment process, which was successfully managed. In all of the children treated this way, the underlying disease no longer progresses, allowing them to develop and reach developmental milestones that would not have been possible without treatment [7]. Due to safety concerns linked to high intravenous doses, onasemnogene abeparvovec was only accessible for patients under 2 years old in the US. Nonetheless, additional clinical studies have been conducted to assess the safety and effectiveness of delivering the treatment directly into the CSF [50]. These trials have confirmed that intrathecal administration of onasemnogene abeparvovec is safe and well-tolerated in older SMA patients. By employing fixed, low doses given intrathecally, this method could offer a feasible treatment option for heavier patients who cannot receive weight-based intravenous dosing [52].

In 2018, 2020 and 2021, we sent our first patients with inborn errors of metabolism abroad for experimental gene therapy; the first with mucopolysaccharidosis (MPS) type I (the first gene-treated child with this diagnosis in the world), and the second and third with MPS type IIIa [1, 2]. The treatment was successful in all three of them and they are now being regularly monitored in Slovenia, in collaboration with both study Centres. The child with MPS I underwent ex vivo gene therapy with lentiviral vector-treated haematopoietic stem cell transplantation at a specialised centre in Italy. The second and third children received a single dose of in vivo gene therapy with the AAV9 vector at a study centre in Spain. So far, the disease course has been favourable, but long-term outcomes will have to wait several years [1, 2].

In 2019, a 10-month-old patient from Slovenia with metachromatic leukodystrophy (MLD) in the asymptomatic stage, diagnosed after the disease was confirmed in his older sibling with the symptomatic late infantile form of the disease, was among the first patients worldwide to receive gene therapy within the scope of a clinical study at an Italian referral medical centre. The transplantation of autologous cryopreserved bone marrow CD34 + cells, transduced ex vivo with a lentiviral vector encoding the human *ARSA* complementary DNA after a conditioning regimen with intravenous busulfan, was performed. There were no major complications during the acute phase of treatment, and after more than 4 years of follow-up by the clinical study centre in Italy and concurrently by UCH, the treatment can be deemed very successful. Apart from some degree of peripheral sensorimotor neuropathy and consequential mild gait impairment, the patient remains without any cognitive or other neurological deficits. Following his treatment, gene therapy for MLD received EMA approval in 2020 [65].

In 2019, when CAR-T lymphocyte therapy was only available in highly specialised centres in Europe, we sent the first Slovenian child abroad to receive such treatment. In April 2020, having obtained the necessary certificates of competence, we administered CAR-T lymphocyte therapy for the first time in Slovenia to a child with refractory acute lymphoblastic leukaemia. This treatment achieved remission in a case that had been resistant to conventional intensive chemotherapy. Since the initial treatment, five additional children have received this therapy at the Department of Haemato-Oncology of the UCH. Four of these patients are alive and continue to maintain complete remission. The adult CAR-T cell program at the Department of Hematology at the University Medical Centre Ljubljana has been ongoing since 2020. During this period, we referred eighteen patients with DLBCL for treatment with tisa-cel, and sixteen received CAR-T cell therapy. One patient experienced CAR-T cell production failure, while another patient’s condition deteriorated during bridging therapy. Treatment outcomes align with published data for tisa-cel, with overall survival in our patient group ranging between 40 and 50% [66]. Anti-BCMA CAR-T cell treatment for multiple myeloma is unavailable in Slovenia. However, since 2023, we have been referring our patients to Germany for treatment with ide-cel. Three patients have been referred, and two were deemed eligible, both undergoing CAR-T cell therapy and remaining in remission at the last follow-up [67].

In addition to the cases mentioned above, according to our data, several other children from Slovenia have been treated with gene therapy abroad since 2019 and are being monitored at the University Medical Centre Ljubljana (with diagnoses of MLD and epidermolysis bullosa).

### Development of clinical infrastructure for the management and gene therapy of rare diseases

In Slovenia, we have developed rapid and accurate genomic diagnostics for rare diseases, which are conducted at the Clinical Institute of Special Laboratory Diagnostics of UCH. This diagnostic capability serves as a prerequisite for gene therapy. We have established our own rapid and reliable genomic diagnostics at the UCH. In 2023, we also established the Register of Rare Non-Malignant Diseases (managed by the UCH), developed under the auspices of the Ministry of Health of the Republic of Slovenia and the National Institute of Public Health). Through collaboration with all key stakeholders and experts in this field, this register provides us with a much better understanding of the epidemiological aspects of rare diseases in Slovenia. Furthermore, we have also established the National contact point for rare diseases (accessible at the link: https://www.redkebolezni.si/en/national-contact-point-for-rare-diseases/), where patients can obtain initial information and guidance. All these activities are carried out within the framework of the Centre for Rare Diseases at the UCH, established in 2023 [13, 68, 69].

### CTNNB1 syndrome as an example of the possibility of the development of gene therapy through a patient cantered network

CTNNB1 syndrome is a neurodevelopmental disorder caused by various mutations that lead to loss of function in one allele of the CTNNB1 gene. The disorder has so far been diagnosed in approximately 400 patients worldwide, three of whom are in Slovenia [12, 70]. When Urban, a one-year-old boy, was diagnosed with a CTNNB1 gene defect at the Department of Paediatric Neurology of UCH his parents expressed their desire for their son to receive gene therapy. Even though such therapy did not exist at that time, nor were there any preclinical studies testing such therapy, they were determined to do everything possible to potentially enable access to such treatment for their son. The parents established the CTNNB1 Foundation (accessible at the link: https://ctnnb1-foundation.org/), through which they began raising funds for the first steps of preclinical research. Initially, a crucial research partner was found in the group led by Prof. Dr. Roman Jerala at the NIC. Additionally, we collaborated with the research group of Assoc. Prof. Dr. Leszek Lisowski from the Children’s Medical Research Institute in Australia. The latter began developing gene therapy using an AAV9 vector on Urban’s cells and organoids, while Prof. Jerala’s group started exploring alternative treatment technologies and supporting preclinical drug testing in the Laboratory for in vivo experiments, led by Dr. Duško Lainšček at the NIC. Within the Department of Paediatric Neurology of UCH, we conducted a large cross-sectional study (ClinicalTrials.gov: NCT04812119), inviting all known patients with CTNNB1 syndrome worldwide to better understand the connection between genetic changes and the clinical presentation of the disease. We aimed to identify the primary challenges faced by these patients and determine which parts of the central nervous system potential new drugs should target. Concurrently, we secured a research project grant from the Slovenian Research and Innovation Agency to alleviate the financial burden on parents during drug development. Once candidate drugs were identified through work on cells and cell organoids, they were tested on a mouse model of CTNNB1 syndrome created specifically for this purpose. The results of the preclinical research were promising, so in the autumn of 2023, we presented the findings to Viralgen (Spain), a company capable of manufacturing the drug by the highest quality standards in a manner suitable for use in patients. Many more challenges lie ahead to confirm the safety and efficacy of the drug. Simultaneously, we are preparing a CTNNB1 natural history study and a clinical trial with the assistance of researchers in Oxford (United Kingdom), through which the drug could be appropriately administered to the first patients. We hope to conduct the clinical trial at the UCH. Parallel discussions have been ongoing with regulatory agencies, such as EMA, and the Agency for Medicinal Products and Medical Devices of the Republic of Slovenia, to ensure that the potential drug will meet al.l safety and quality requirements for an orphan drug designation (Miroševic S, et al. Paving the way towards treatment solutions for CTNNB1 syndrome. [Submitted].).

### Immune gene therapy for cancer

One method of delivering genetic material into cells for gene therapy purposes is electroporation. This process destabilizes the cell membrane, allowing the entry of a non-viral vector, usually plasmid DNA encoding the therapeutic transgene, into tumour cells. These cells then become producers of this transgene, which encodes the desired therapeutic protein [9]. Immunotherapy has emerged as a powerful tool in the fight against cancer. Immune checkpoint inhibitors (ICIs) have garnered widespread attention as standalone therapies or in combination with other local ablative therapies. To further enhance the efficacy of immunotherapy, researchers at the Institute of Oncology in Ljubljana have developed a plasmid encoding IL-12 for use as adjuvant therapy for tumours of different histologies. Under the SmartGene.Si project, we constructed a plasmid DNA encoding IL-12, a cytokine with immunomodulatory and antitumor properties. Collaborating with industry partners, a good manufacturing practice-compliant production process was established for this plasmid, ensuring its suitability for clinical use. Adhering to European Union regulations for advanced therapy medicinal products and antitumor drugs, we engaged with the EMA and its Committee for Advanced Therapies (CAT) to seek guidance on non-clinical testing and clinical trial design for our product. Based on EMA’s recommendations, we conducted a comprehensive battery of non-clinical safety and efficacy studies for the plasmid. The results of our in vitro studies have been published, while the in vivo findings are currently undergoing the publication process [10, 11]. Following EMA’s CAT recommendations, we prepared a comprehensive clinical protocol that received approval from the Agency for Medicinal Products and Medical Devices of the Republic of Slovenia and the National Ethics Committee [9]. In early November 2021, at the Institute of Oncology Ljubljana in collaboration with the Department of Otorhinolaryngology and Cervicofacial Surgery of the University Medical Centre Ljubljana, we commenced a Phase I clinical trial involving intratumoral electroporation of a plasmid encoding IL-12 [6]. The Phase I clinical trial has been completed, demonstrating the safety and feasibility of the treatment. In addition, the starting dose of plasmid encoding IL-12 for phase II studies has been established. The results are currently being prepared for publication. Furthermore, we are planning a Phase II clinical trial to evaluate the combined use of our IL-12 immune gene therapy in combination with ablative treatments such as radiotherapy or electrochemotherapy.

### Challenges related to gene therapy

Gene therapy, while holding immense promise for treating various genetic disorders, is not without its ethical challenges and unresolved questions. One of the primary concerns surrounding gene therapy is its high cost. However, in some cases, the cost is lower than the long-term treatment of the same patients with other drugs, such as enzyme replacement therapy or clotting factor treatment in haemophilia. Moreover, as gene therapy technology matures and competition increases, the cost is expected to decrease, making it more accessible to a broader patient population [71–73]. For SMA, we have already reached a point where early treatment of children identified through newborn screening not only provides significantly better quality of life for the patient but is also cheaper than treating late complications of the disease and disability [74, 75]. The problem of sufficient production and, especially in the case of ex vivo approaches, distribution of gene therapy will need to be addressed [35]. Academic-led initiatives may help counterbalance commercial barriers by advancing non-commercial development pathways, especially for rare diseases, as emphasized by Bueren and Auricchio, who advocate for publicly supported, sustainable gene therapy models [76].

For now, we cannot treat diseases caused by alterations in multiple genes or chromosomal abnormalities. The short time that gene therapies have been around also means that the long-term safety and efficacy of gene therapy are not yet clear, although modern approaches show great promise in this regard. Moreover, it is likely to remain possible for some time to help only specific groups of patients with rare diseases. Drug development is expensive and challenging, and there is no guarantee that individual progress in development will lead to a successful cure [14]. Encouragingly, the CTGCT is being established under the auspices of the NIC, following the success in the Teaming for Excellence call of the European Commission. This will enable some preclinical development steps to be carried out domestically, facilitating faster access for Slovenian patients to clinical trials of advanced therapies.

## Conclusion

Gene therapy represents a significant milestone for patients with rare diseases. The last decade has brought great progress in this field, and gene therapy has been successfully utilized in the treatment of the first Slovenian patients in recent years. It is expected that in the next decade, there will be even more significant advancements in this area, which we must consider when planning and organizing activities in this field, particularly regarding early detection (i.e., in newborns) through screening, especially for diseases that are already treatable. Encouragingly, the development of gene therapy has brought together researchers in the field of biomedicine, clinicians, and patient organizations in various areas, which is essential for the success of such complex approaches. However, Slovenian medicine must continue to align with the most advanced developments in this field, allowing us to promptly implement innovations in practice. Currently, access to innovative therapies in Slovenia is at a high level. It is imperative for clinicians, researchers, and other stakeholders to collaborate closely to develop appropriate regulations that will broaden access to future gene therapies.

## Data Availability

Not applicable.

## Abbreviations

AAV9: Adenovirus-associated viral vector 9
BCMA: B-cell maturation antigen
CAR: Chimeric antigen receptor
CAT: Committee for advanced therapies
CSF: Cerebrospinal fluid
CTGCT: Centre for the technologies of gene and cell therapy
DLBCL: Diffuse large B-cell lymphoma
EMA: European medicines agency
ICIs: Immune checkpoint inhibitors
IL-12: Interleukin 12
LNP: Lipid nanoparticle
MLD: Metachromatic leukodystrophy
MPS: Mucopolysaccharidosis
NIC: National institute of chemistry
OTC: Ornithine transcarbamylase
RNAi: RNA interference
SCID-X1: X-linked severe combined immunodeficiency
SMA: Spinal muscular atrophy
UCH: University children's hospital of the university medical centre Ljubljana
